# Characterization of Human CD39^+^ Th17 Cells with Suppressor Activity and Modulation in Inflammatory Bowel Disease

**DOI:** 10.1371/journal.pone.0087956

**Published:** 2014-02-05

**Authors:** Maria Serena Longhi, Alan Moss, Aiping Bai, Yan Wu, Huang Huang, Adam Cheifetz, Francisco J. Quintana, Simon C. Robson

**Affiliations:** 1 Division of Gastroenterology, Department of Medicine, Beth Israel Deaconess Medical Center, Harvard University, Boston, United States of America; 2 Institute of Liver Studies, King’s College London School of Medicine at King’s College Hospital, London, United Kingdom; 3 Center for Neurologic Diseases, Brigham and Women's Hospital, Harvard Medical School, Boston, United States of America; Cincinnati Children's Hospital Medical Center, University of Cincinnati College of Medicine, United States of America

## Abstract

Induced regulatory T-cells (iT-reg) and T helper type 17 (Th17) in the mouse share common CD4 progenitor cells and exhibit overlapping phenotypic and functional features. Here, we show that human Th17 cells endowed with suppressor activity (supTh17) can be derived following exposure of iT-reg populations to Th17 polarizing conditions. In contrast to “pathogenic” Th17, supTh17 display immune suppressive function and express high levels of CD39, an ectonucleotidase that catalyzes the conversion of pro-inflammatory extracellular nucleotides ultimately generating nucleosides. Accordingly, supTh17 exhibit nucleoside triphosphate diphosphohydrolase activity, as demonstrated by the efficient generation of extracellular AMP, adenosine and other purine derivatives. In addition supTh17 cells are resistant to the effects of adenosine as result of the low expression of the A2A receptor and accelerated adenosine catalysis by adenosine deaminase (ADA). These supTh17 can be detected in the blood and in the lamina propria of healthy subjects. However, these supTh17 cells are diminished in patients with Crohn’s disease. In summary, we describe a human Th17 subpopulation with suppressor activity, which expresses high levels of CD39 and consequently produces extracellular adenosine. As these uniquely suppressive CD39^+^ Th17 cells are decreased in patients with inflammatory bowel disease, our findings might have implications for the development of novel anti-inflammatory therapeutic approaches in these and potentially other immune disorders.

## Introduction

CD4^+^CD25^high^FOXP3^+^ regulatory T-cells (T-reg) are central to the maintenance of immune homeostasis [1]–[4]. T-reg prevent or even reverse experimental autoimmunity, and T-reg cellular defects have been observed in association with various autoimmune disorders, such as those associated with vascular thrombophilia as in inflammatory bowel disease [1]–[3]. T-reg exert suppressive function by releasing inhibitory cytokines, such as IL-10 [5], [6], TGF-β [7], [8] and IL-35 [9]; by cytolysis, mainly mediated by granzyme B [10]; by modulating the maturation and the antigen presenting ability of dendritic cells [11]; or by metabolic disruption either by depriving of IL-2 effector cells [12] or by hydrolyzing pro-inflammatory ATP into immunomodulatory adenosine, secondary to the specific co-expression of CD39 and CD73 ectonucleotidases by such cells [13], [14].

In contrast, T helper type 17 lymphocytes (Th17) are an effector subset that develops independently of Th1 and Th2 cell lineages. Th17 cells drive inflammatory and autoimmune conditions in both mice and humans and have been linked to intestinal inflammation [15], [16]. CD4^+^ T-cells can be differentiated into Th17 cells when exposed to TGF-β in combination with IL-6 or IL-21 in mice and to IL-6, TGF-β and IL-1β in humans, or into induced (i)T-reg under the influence of TGF-β [15], [16]. Additional studies have shown that, in addition to TGF-β, other factors including IL-2 [17], [18] and anti-CD3/anti-CD28 [19] play a role in iT-reg generation, even after a short stimulation period [19]. iT-reg and Th17 cells, however, may not be terminally differentiated and iT-reg in particular show phenotypic and functional plasticity [20].

Using genetic lineage tracing of Foxp3 T-reg, Zhou and colleagues observed that a significant proportion of Foxp3^+^ cells undergo down-regulation and in some cases loss of Foxp3 expression is noted [21]. These ‘ex-Foxp3’ cells display an effector memory cell phenotype, produce pro-inflammatory cytokines and are numerically increased in experimental autoimmune diabetes [21]. Moreover, exposure of T-reg to IL-6 can down-regulate both Foxp3 and IL-17 expression, suggesting that T-reg may be ‘subverted’ to Th17-like cells [22]. In addition, it has been reported that T-reg can further acquire effector properties - i.e. IFNγ production - when cultured in the presence of IL-12 [23]. These ‘Th1-like’ T-reg show diminished suppressive activity that can be only partially reversed by blockade of IFNγ or IL-12 removal [23].

The stimulation of naïve T-cells with TGF-β and IL-6 triggers IL-17 production but it also induces the expression of IL-10, limiting the pathogenic potential of these cells [24]. Indeed, additional studies have reported that IL-17^+^ T-cells can limit tissue damage during inflammation [25], [26]. In experimental murine tumor settings, it has been demonstrated that CD39 and CD73 expressed by ‘suppressor’ Th17 cells (supTh17) suppress tumor-specific immunity [27]. Whether comparable human supTh17 cells exist has been unexplored to date.

CD39 hydrolyses ATP and ADP into AMP, which is then converted into adenosine by CD73. The regulatory properties of CD39 were initially noted in studies conducted on CD39^null^ mice in which an enhanced production of IFNγ, IL-1β, IL-6 and TNF-α was found [28], [29]. CD39 and CD73 expression on murine T-reg is required for the suppressive function of these cells, which results from the production of adenosine [4]. Accordingly, T-reg isolated from CD39^null^ mice are unable to block allograft rejection in adoptive transfer studies [13].

Expression of CD39 has been reported on human T-reg in parallel to FOXP3 and low levels of CD127 [30], [31]. Human T-reg do not co-express high levels of CD73 with CD39 in contrast to murine counterparts. Thus AMP conversion to adenosine by human CD39^+^ T-reg is thought to result from paracrine mechanisms by the presence of CD73 on target or neighboring cells [31]. Regardless of the molecular mechanism involved, it has been shown that human CD39^+^ T-reg exert preferential suppression on CD4 target cell IL-17 production [32].

Defective numbers of CD39^+^ T-reg have been reported in patients with multiple sclerosis, autoimmune hepatitis [33] and CD39 polymorphisms linked to low-level CD39 expression have also been described in Crohn’s disease [30], [32], [34]. Recent studies have shown that in addition to T-reg, CD39 is also expressed on a subset of memory cells with effector function [35]. Although this expression of CD39 by human T-reg has been reported and putative roles dissected, the demonstration and relevance of specific CD39 expression by human Th17 cells has been unexplored to date.

We describe here a population of human supTh17 cells that in contrast to prototypic pathogenic Th17 display high levels of both CD39 and FOXP3 and exhibit immune suppressive properties. Our new observations also provide mechanistic insights into the development of supTh17 and indicate the role of CD39 and purinergic immunomodulation. The pathophysiological relevance of these cells is supported by the detection of decreased frequencies of CD39^+^ supTh17 cells in both peripheral blood and lamina propria of patients with Crohn’s disease, an illness characterized by manifestations of unfettered intestinal inflammation.

## Materials and Methods

### Subjects

Peripheral blood mononuclear cells (PBMCs) were isolated from platelet-depleted blood (leukofilters) obtained from 68 healthy blood donors (Blood Donor Center at Children’s Hospital, Boston, MA). PBMCs were also obtained from 25 patients with Crohn’s disease, recruited from the Gastroenterology Division, Beth Israel Deaconess Medical Center (BIDMC), Boston MA. Of these patients, 11 were studied during active disease (median Harvey Bradshaw Index, HBI: 8, range 2 to 25) while 14 were in remission (median HBI: 0, range 0–12). At the time of investigations, 11 patients were receiving infliximab, 2 were on steroids and 2 on immunomodulatory drugs.

### Ethics Statement

The study was approved by BIDMC Institutional Review Committee. Written consent was obtained from all study participants.

### Cell Purification

PBMCs were obtained by density gradient centrifugation on Ficoll-Paque (GE Healthcare, Uppsala, Sweden). Cell viability, determined by Trypan Blue exclusion, exceeded 98%. Lamina propria mononuclear cells (LPMCs) were isolated from freshly biopsied colonic tissue. The tissue was initially washed with PBS, cut into small sections and incubated in calcium and magnesium-free HBSS containing 4 mM EDTA and 1 mM dithiothreitol at 37°C for 15 min. Epithelia were removed by discarding the supernatants. This procedure was repeated three times. The tissue was then minced, resuspended in RPMI 1640 containing 10% FCS, 400 U/ml collagenase D and 0.01 mg/ml DNase I, and then incubated at 37°C for 1.5 hour with pipetting every 30 min. The digested tissue was filtered and centrifuged at 600×*g* for 7 min. Collected cells were pelleted, resuspended in PBS 1% FCS and stained as indicated below.

### Cell Sorting and Culture

CD4^mem^ and CD4^naive^ cells were sorted as CD4^+^CD45RO^+^ and CD4^+^CD45RA^+^ from PBMCs using a BD FACSAria (BD Biosciences, San José, CA) (purity higher than 98%). Cells were cultured in complete RPMI 1640 medium (Invitrogen, Carlsbad, CA) supplemented with 2 mM L-glutamine, 100 U/ml penicillin, 100 µg/ml streptomycin, 1% non-essential amino acids and 10% FCS and exposed for 3 days to Th17 polarizing conditions (Figure S1), i.e. IL-6 (50 ng/ml)+IL-1β (10 ng/ml)+TGF-β (3 ng/ml) [36]–[38] and anti-CD3/anti-CD28 T-cell expander (bead/cell ratio: 1/50) (Dynal Invitrogen). In some experiments cells were exposed to additional Th17 polarizing conditions, namely IL-6+IL-1β+IL-23 (20 ng/ml) or IL-6+IL-1β+IL-23+TGF-β. All cytokines were from R&D Systems (Minneapolis, MN). Cells were then stimulated for 4 days in the presence of iT-reg polarizing conditions consisting of high concentration IL-2 (300 U/ml) and T-cell expander (bead/cell ratio: 1/2) [39], [40] and then re-exposed to the same Th17 polarizing conditions indicated above for additional 3 days (Figure S1). Cells obtained after exposure to Th17 and iT-reg polarizing conditions are referred to as Th17 and iT-reg; cells obtained after iT-reg exposure to Th17 driving conditions are indicated as supTh17 (Figure S1). Functional properties of Th17, iT-reg and supTh17 are described in the ‘Results’ section.

### Flow Cytometry

Cell phenotype was assessed by 6-colour flow cytometry following cell incubation with FITC, PE, PE-Cy7, Pacific blue (PB), APC and APC-Cy7-conjugated anti-human antibodies to: CD4 (clone#: OKT4), CD45RO (clone#: UCHL1), CD45RA (clone#: HI100), CD25 (clone#: BC96), CD26 (clone#: BA5b), CD39 (clone#: A1), CD73 (clone#: AD2), CCR6 (clone#: G034E3) (all from Biolegend, San Diego, CA) and IL-23R (R&D Systems, clone#: 218213). Frequency of FOXP3, RORC and Stat-3 positive cells was assessed by intracellular staining following cell fixation and permeabilization with Cytofix/Cytoperm (BD Biosciences) and incubation with PB, APC and PE-conjugated anti-human FOXP3 (Biolegend, clone#: 206D), RORC (eBioscience, San Diego, CA; clone#: AFKJS-9) and Stat-3 (BD Bioscience, clone #: 49/p-Stat-3). Frequency of cytokine-producing cells was determined after exposure to phorbol 12-myristate 13-acetate (PMA, 10 ng/ml, Sigma-Aldrich) and Ionomycin (500 ng/ml) for 60 minutes and to Brefeldin A (20 µg/ml, Sigma-Aldrich) for additional 5 hours. Staining was carried out using PE, PB, and APC-conjugated anti-human antibodies to IFNγ (Biolegend, clone#: 45.B3), IL-17A (Biolegend, clone#: BL168), IL-10 (BD Biosciences, clone#: JES3-19F1), IL-2 (BD Bioscience, clone#: MQ1-17H12) and IL-22 (eBioscience, clone#: IL22JOP). Isotype controls were from BD Biosciences. Cells were acquired on a BD LSRII (BD Biosciences) and analyzed using BD FACSDiva software. 3–5×10^4^ events were acquired for each sample. Positively stained cell populations were gated based on unstained, single stained and isotype stained controls. Effect of adenosine (Sigma-Aldrich, St. Louis, MO) on Th17, iT-reg and supTh17 phenotype was assessed in parallel experiments. Adenosine was added at 50 µM to memory CD4 cells at baseline; after 3 days when exposing cells to iT-reg polarizing conditions; and after additional 4 days when re-stimulating cells in the presence of Th17 skewing conditions. Controls consisted of cultures in the absence of adenosine.

### *In vitro* Suppression Assay

The ability of Th17, iT-reg and supTh17 to control target cell proliferation and effector cytokine production was evaluated following 4-day co-culture with CD4 responder cells. Following 24 hour resting in cytokine and bead-free medium, Th17, iT-reg and supTh17 were added at 1/8 ratio to autologous CD4 target cells (2.5×10^4^ cells/well) previously exposed to IL-2 (30 U/ml) and T-cell expander (bead/cell ratio: 1∶2) for 5 to 7 days. The 1∶8 ratio was selected because capable of exerting a detectable regulatory function in preliminary experiments where ratios of 1∶16, 1∶8, 1∶4 and 1∶2 were compared as these putatively reflect pathophysiological proportions between suppressor and effector lymphocytes. Parallel cultures of CD4 responder cells and of Th17, iT-reg and supTh17 on their own were performed under identical conditions. All experiments were performed in duplicates. After 4 days, cultures were pulsed with 0.25 µCi/well ^3^H-thymidine and harvested 18 hours later using a cell harvester (Tomtec, Hamden, CT). Incorporated thymidine was measured by liquid scintillation spectroscopy. In preliminary experiments, inhibition of CD4 target cell proliferation in the absence and presence of suppressor cells was also analyzed using carboxy fluorescein succinimidyl ester (CFSE) staining. As CFSE- and ^3^H-thymidine-based assays gave comparable results, given the requirement for fewer cells, ^3^H-thymidine was used to measure proliferation in subsequent experiments. The ability of Th17, iT-reg and supTh17 cells to control the production of IFNγ and IL-17 by target cells was determined by intracellular cytokine staining after 4-day co-culture as detailed above. The effect of adenosine on Th17, iT-reg and supTh17 ability to suppress was tested in parallel experiments.

### Quantitative Real-time PCR

Expression of A1, A2A, A2B and A3 adenosine receptors, and of phosphodiesterases (PDE) 4A and PDE4B was determined by real-time PCR. Total RNA was extracted from 2–3×10^5^ cells using TRIzol reagent (Invitrogen) and mRNA was reverse transcribed using iScript cDNA Synthesis kit (Bio-Rad Laboratories, Hercules, CA) according to the manufacturer’s instructions. Sequences of adenosine receptors were as previously described [41]. PDE primer sequences were as follows:

PDE4A: Forward 5′ ACACAGCAGTGACGCTAATCCAGA 3′

Reverse 5′ ATTCACTGGAGGAGGTGGCTCAAA 3′

PDE4B: Forward 5′ ACAGCCTGATGCTCAGGACATTCT 3′

Reverse 5′ AAACTTCTCCATCAGACCCTGGCA 3′

PCR amplification conditions were as previously reported [41]. Samples were run on a Stratagene MX3005P (Agilent Technologies, Santa Clara, CA) and results were analyzed by matched software and expressed as relative quantification. Relative gene expression was determined by normalizing to human β-actin (primer sequence as previously reported [41]).

### Immunoblot Analysis

5×10^5^ cells were lysed in ice-cold RIPA buffer, containing 1% NP-40, 0.25% sodium deoxycolate, 50 mM Tris-HCl and 150 mM NaCl and supplemented with Complete Proteinase Inhibitor Cocktails (Roche Diagnostics, Indianapolis, IN) and Phosphatase Inhibitor Cocktails (Sigma-Aldrich). Following 30 minutes incubation on ice, samples were spun at 14,000×*g* for 30 minutes. Supernatants (containing total cell lysates) were collected and total protein concentration determined using Bio-Rad *Dc* protein assay reagent (Bio-Rad Laboratories) using bovine serum albumin as standard. Following protein denaturation with SDS, cell lysates were separated on a 4–12% Criterion XT Bis-Tris SDS-PAGE (Bio-Rad Laboratories). 10 µg of protein were loaded per lane. Gels were run for 20 minutes at 80 V and then at 110 V for additional 80 minutes. Proteins were then transferred onto PVDF membranes (Immobilon-P, Millipore, Billerica, MA) by semi-dry electroblotting and subsequently incubated in blocking buffer containing 2.5% skimmed milk. Following 60 minutes, mouse anti-adenosine deaminase (ADA) antibody (Abcam, Cambridge, MA) was applied at 1 µg/ml. Following overnight incubation membranes were incubated for 60 minutes with HRP-labeled goat anti-mouse (Thermo Scientific, Rockford, IL) at 1/50,000. Bands were visualized using SuperSignal West Femto Maximum Sensitivity Substrate (Thermo Scientific) according to the manufacturer’s instructions. For immunoblot normalization, the same membranes were stripped (using a buffer containing 15 g glycine, 1 g SDS and 10 ml Tween20) and re-probed with mouse anti-human β-actin (Abcam) at 1/20,000 and subsequently with a HRP-labeled goat anti-mouse polyclonal antibody at 1/20,000. ADA band density was determined using Scion Image Processing Program (Release Beta 4.0.2).

### Ectonucleotidase Enzymatic Activity Analysis

Thin layer chromatography (TLC) was performed as previously described [13], [42]. 3×10^5^ Th17, iT-reg and supTh17 were incubated with 2 mCi/ml [C^14^]ADP (GE Healthcare Life Sciences) in 10 mM Ca^2+^ and 5 mM Mg^2+^. Then, 5 µl aliquots, collected at 5, 10, 20, 40 and 60 minutes, were analyzed for the presence of [C^14^]ADP hydrolysis products by TLC and applied onto silica gel matrix plates (Sigma-Aldrich). [C^14^]ADP and the radiolabeled derivatives were separated using an appropriate solvent mixture as previously described [43].

### Statistical Analysis

Results are expressed as mean±SEM (obtained from at least 5 subjects per group and from at least 3 independent *in vitro* experiments). Smirnov goodness of fit test was performed to test the normality of variable distribution. Paired and unpaired Student’s *t* test were used for comparing normally distributed data; Wilcoxon’s rank sum test and Mann Whitney test were used for non-normally distributed data. ANOVA repeated measures or one-way ANOVA, followed by Tukey’s multiple comparisons test, was used to compare the means of multiple samples. For all comparisons a *P* value ≤0.05 was considered significant. Statistical analysis was performed using SPSS version 19.0.

## Results

### supTh17 are Phenotypically Different from Prototypic Th17 Cells and Display Regulatory Function

In order to first investigate whether human Th17 cells can acquire regulatory functions per se, we activated CD4^+^CD45RO^+^ memory (CD4^mem^) and CD4^+^CD45RA^+^ naïve (CD4^naive^) T-cells under Th17 polarizing conditions. Next, we exposed these cells to iT-reg polarizing conditions. Finally, to evaluate the stability of the polarized T-cells, we re-activated them in the presence of Th17 skewing conditions, as detailed in Methods (see also Figure S1). In these studies, Th17 polarizing conditions consisted of 3-day exposure to IL-6, IL-1βand TGF-β, a cytokine cocktail previously shown to result in the efficient differentiation of IL-17 producing cells in humans [36]–[38], [44], and to low dose anti-CD3/anti-CD28. Further, iT-reg polarizing conditions consisted of 4-day stimulation in the presence of high concentration IL-2 and anti-CD3/anti-CD28, shown to be particularly effective at inducing high numbers of effective iT-reg [39], [40], [44].

**Figure 1:**
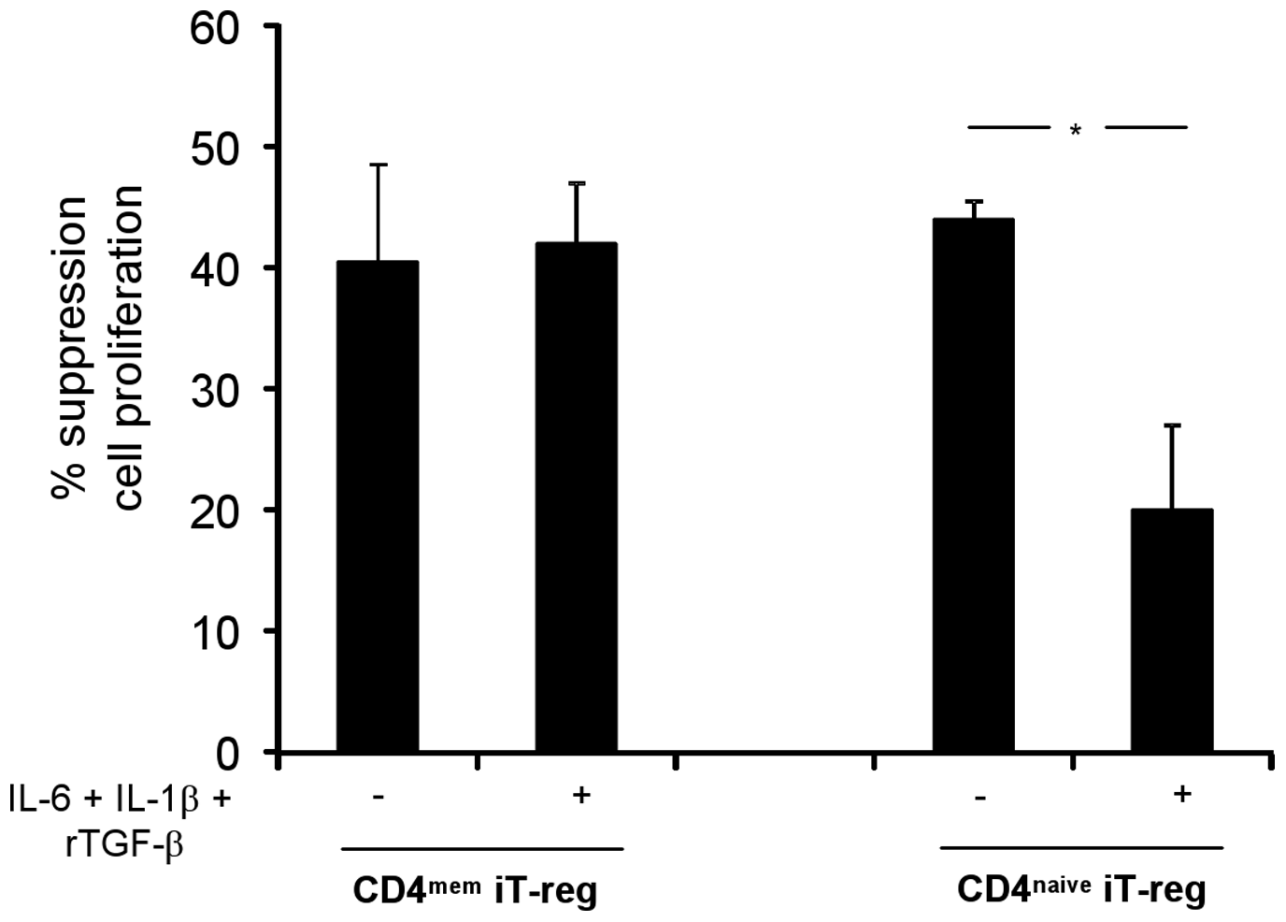
Suppressor ability of iT-reg derived from CD4^+^CD45RO^+^ memory (CD4^mem^) and from CD4^+^CD45RA^+^ naïve (CD4^naive^) cells. The ability of iT-reg obtained from CD4^mem^ and CD^naive^-derived Th17 cells was evaluated after 4-day co-culture by ^3^H-thymidine incorporation in 5 healthy subjects. Mean (+SEM) percentage suppression of CD4 effectors by CD4^mem^ or CD4^naive^ iT-reg before and after exposure to IL-6, IL-1β and rTGF-β. CD4^mem^ but not CD4^naive^ iT-reg maintain their suppressor ability after exposure to Th17 driving cytokines. *P≤0.05.

We found that iT-reg obtained from CD4^mem^-derived Th17 cells had persistent and stable suppressor activity following “re-activation” in the setting of Th17 polarizing conditions (Figure 1). In contrast, iT-reg obtained from CD4^naive^-derived Th17 cells, had lost most of their suppressive ability once re-activated in the presence of Th17 polarizing conditions (Figure 1). Therefore, we focused consequent studies on iT-reg derived from CD4^mem^.

**Figure 2:**
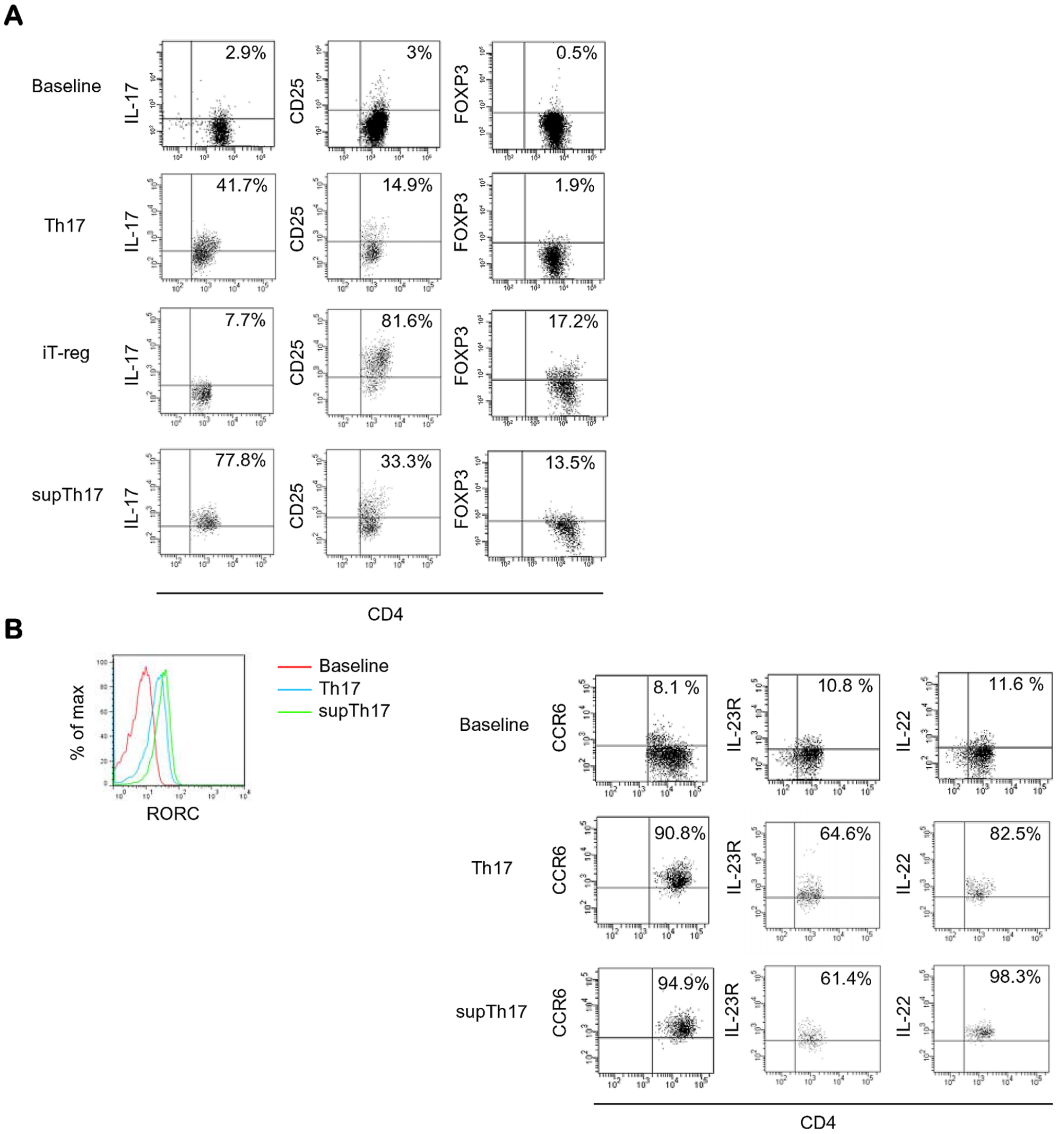
Phenotypic properties of supTh17. Phenotype of CD4^mem^ at baseline and of Th17, obtained from CD4^mem^ after 3-day exposure to IL-6+IL-1β+rTGF-β; iT-reg, obtained following exposure of Th17 to high concentration IL-2 and T-cell expander; and supTh17, obtained upon iT-reg exposure to IL-6+IL-1β+rTGF-β. Cell phenotype was determined in 12 healthy subjects. (A) Representative flow cytometry plots of CD4 (*X* axis) and IL-17, CD25 and FOXP3 (*Y* axis) fluorescence. (B) Representative histogram depicting RORC fluorescence in CD4^mem^ at baseline, Th17 and supTh17; representative flow cytometry plots of CD4 (*X* axis) and CCR6, IL-23R and IL-22 (*Y* axis) fluorescence. Compared to prototypic Th17, supTh17 display higher frequencies of IL-17^+^, FOXP3^+^ and IL-22^+^ lymphocytes, express similar levels of RORC and contain comparable numbers of CCR6^+^ cells.

Figures 2 and S2 illustrate the phenotype of CD4^mem^ cells at baseline; after 3-day exposure to Th17 polarizing conditions; after further 4-day stimulation in the presence of iT-reg polarizing conditions; and then after 3-day re-exposure to Th17 driving cytokines. CD4^mem^ cells at baseline contained low frequencies of IL-17-producing, CD25^+^ and FOXP3^+^ lymphocytes (Figures 2A and S2A–C). Following 3-day exposure to IL-6, IL-1β and TGF-β, CD4^mem^ cells displayed higher numbers of IL-17-producing cells, while maintaining low frequencies of CD25^+^ and FOXP3^+^ lymphocytes (Figures 2A and S2A–C).

Cells obtained following Th17 exposure to iT-reg polarizing conditions displayed a decrease in the number of IL-17^+^ lymphocytes and an increase in the frequency of CD25^+^ and FOXP3^+^ cells (Figures 2A and S2A–C). These cells contained minimal proportions of effector cytokines like IFNγ or IL-2 (Figure S3). After iT-reg exposure to Th17 polarizing conditions, we noted marked increases in the number of cells producing IL-17, decreases in lymphocytes positive for CD25 and frequencies of FOXP3^+^ lymphocytes that were similar to iT-reg although higher than Th17 cells (Figures 2A and S2A–C). When contrasted to prototypic Th17, these supTh17 cells displayed higher expression of RORC, higher numbers of IL-22^+^ lymphocytes and similar proportions of cells positive for CCR6 and IL-23 receptor (IL-23R) (Figure 2B).

**Figure 3:**
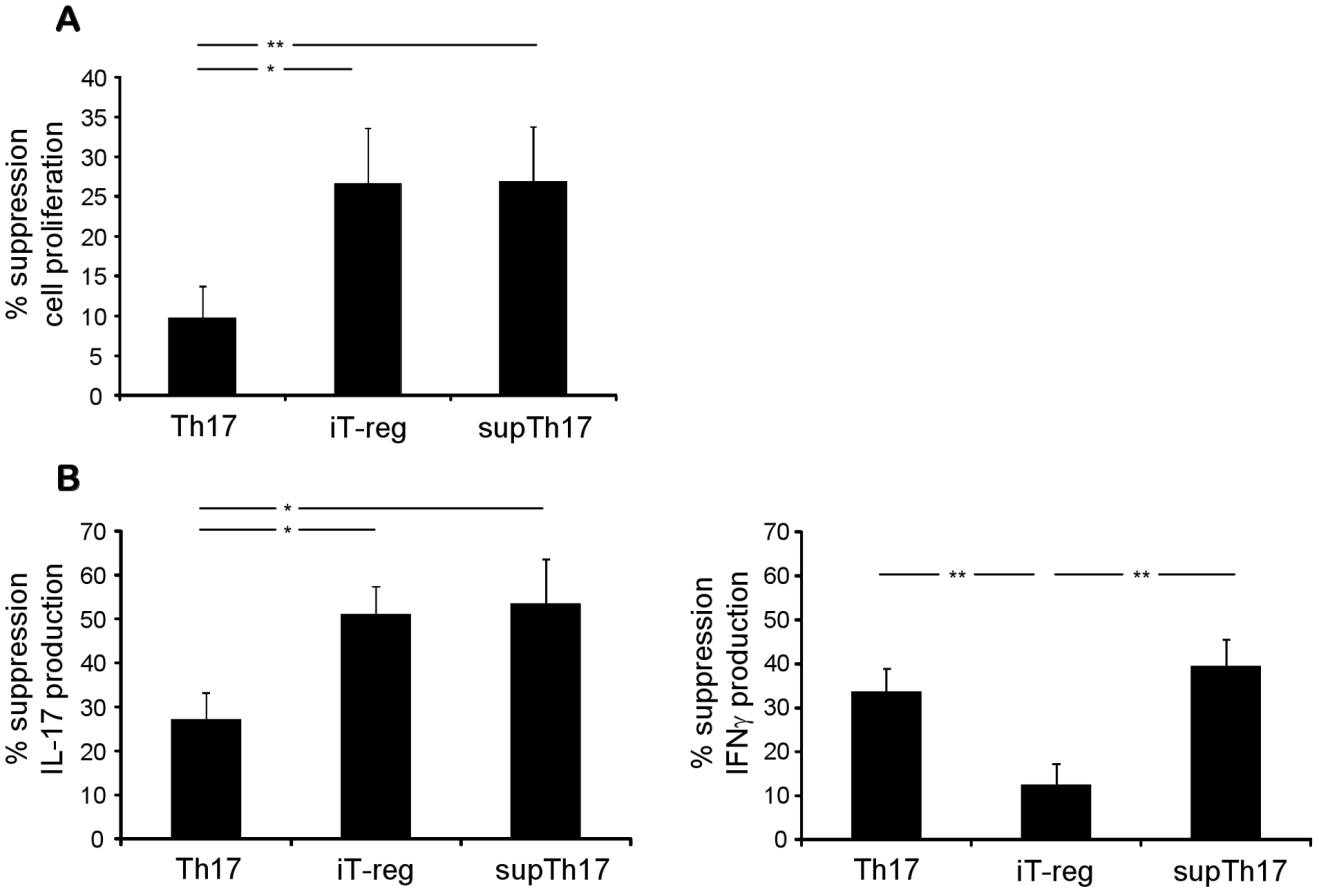
supTh17 suppressive ability. The ability of Th17, iT-reg and supTh17 cells to control CD4 target cell proliferation was evaluated after 4-day co-culture by ^3^H-thymidine incorporation in 10 healthy subjects. (A) Mean (+SEM) percentage inhibition of CD4 effector cell proliferation by Th17, iT-reg and supTh17 cells. (B) The ability of Th17, iT-reg and supTh17 cells to control CD4 target cell IL-17 and IFNγ production was evaluated after 4-day co-culture by intracellular cytokine staining in 10 healthy subjects. Mean (+SEM) percentage inhibition of CD4 effector cell IL-17 and IFNγ production by Th17, iT-reg and supTh17 cells. Compared to prototypic Th17, supTh17 exerted more effective control over CD4 cell proliferation and pro-inflammatory cytokine production. *P≤0.05; **P≤0.01.

When next considering suppressive functions (Figures 3A and S4A), we observed that supTh17 controlled CD4 target cell proliferation in a comparable manner to iT-reg, and more effectively than did prototypic Th17 cells. With regard to suppression of pro-inflammatory cytokine production (Figures 3B and S4B–C), supTh17 effectively controlled IL-17 and IFNγ cytokine production by CD4 effector cells. In contrast, iT-reg, while effectively inhibiting production of IL-17, exerted only weak control over CD4 T-cell IFNγ production.

In summary, supTh17 can be obtained following exposure of CD4^mem^-derived iT-reg to Th17 polarizing conditions. In contrast to prototypic Th17, these cells contain higher frequencies of IL-17 producing and FOXP3^+^ lymphocytes and furthermore display effective and stable suppressive function.

### The supTh17 Cells Express both CD39 and CD73 thereby Generating Adenosine and other Nucleoside Derivatives

**Figure 4:**
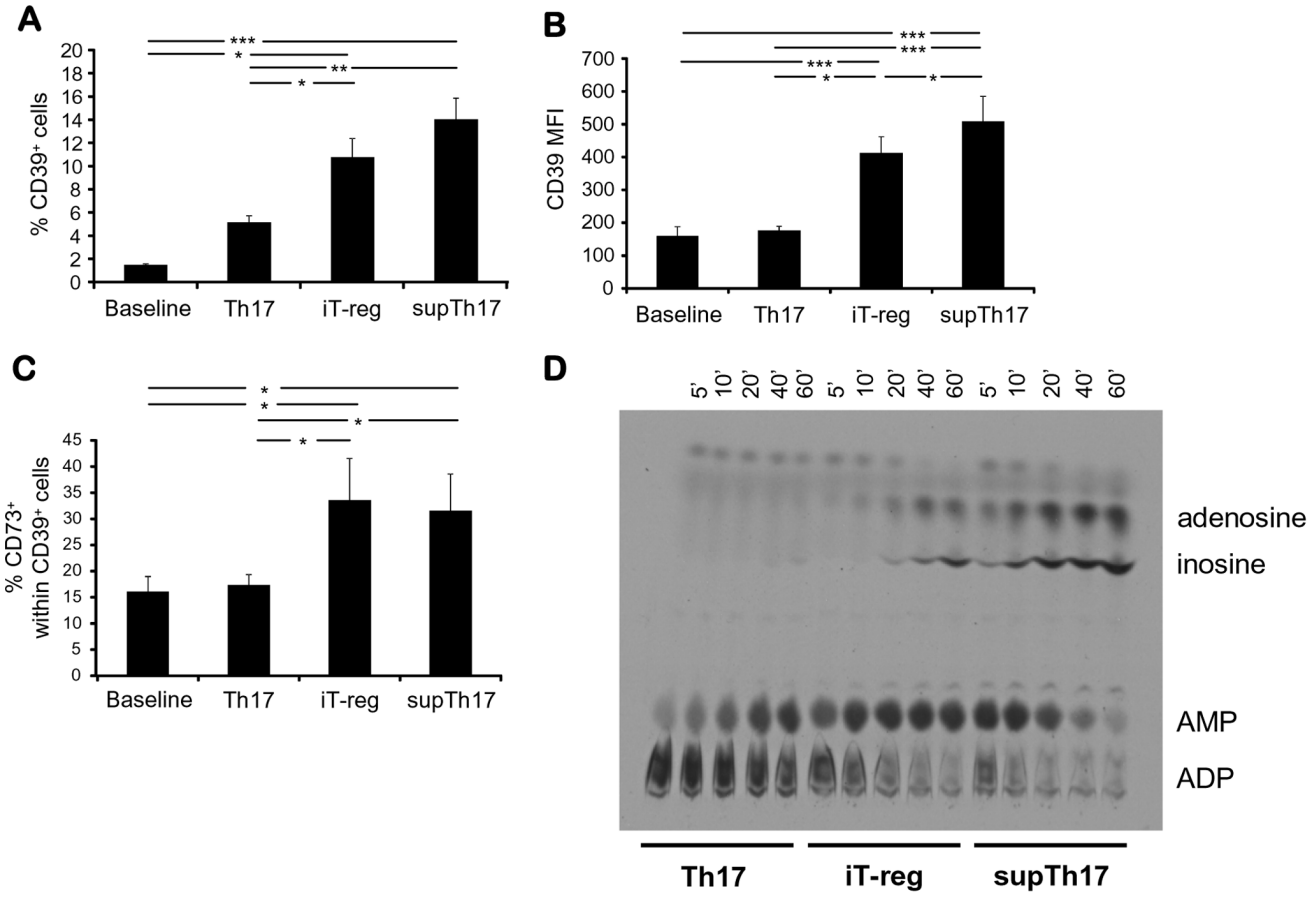
Expression of CD39 and CD73 ectonucleotidases and associated ectoenzymatic activity. (A) Mean (+SEM) frequency of (A) CD39^+^ cells, (B) CD39 mean fluorescence intensity (MFI) and of (C) CD39^+^CD73^+^ cells within CD4^mem^ at baseline and within Th17, iT-reg and supTh17. Results from 12 healthy subjects are shown. *P≤0.05; **P≤0.01; ***P≤0.001. (D) CD39 ADPase enzymatic activity was assessed by TLC following incubation of Th17, iT-reg and supTh17 with [^14^C] radiolabeled ADP substrates. A representative of 3 independent experiments is shown. In accordance with high levels of CD39 and CD73, supTh17 generate AMP, adenosine and its derivative inosine.

Given the regulatory properties displayed by supTh17 and the association between CD39 and immunoregulation [13], [32], we determined the expression of CD39 in supTh17 and compared it with that of CD4^mem^ cells at baseline, Th17 and iT-reg. As shown in Figure 4A and 4B, supTh17 contained the highest frequencies of CD39^+^ cells and displayed the highest CD39 MFI, being therefore clearly distinguishable from prototypic Th17 cells that displayed low numbers of CD39^+^ lymphocytes and low CD39 MFI.

To evaluate whether different Th17 polarizing conditions influence CD39 expression, we obtained Th17 and supTh17 cells upon exposure to IL-6, IL-1β and IL-23 or IL-6, IL-1β, IL-23 and TGF-β. As depicted in Figure S5A, no differences were observed in the frequency of CD39^+^ cells in the presence of different Th17-inducing cytokine cocktails.

We next evaluated the phenotypic properties of CD39^+^ cells within supTh17 and compared with those of CD39^+^ cells within CD4^mem^ at baseline, Th17 and iT-reg (Figures 4C, S5B and S6A–C). supTh17 cells contained proportions of cells positive for CD73 - the ectonucleotidase working in tandem with CD39 to generate adenosine - and for FOXP3 comparable to iT-reg and higher than Th17 cells and CD4^mem^ cells at baseline (Figures 4C, S5B and S6A). No significant differences in the frequencies of IL-10^+^ and RORC^+^ cells were noted between supTh17 and the other cell subsets (Figure S6B–C).

Given the concomitant expression of CD39 and CD73 by both supTh17 and iT-reg, we determined the ability of these cells to generate adenosine. Cell ectoenzymatic activity was assessed by thin layer chromatography (TLC) following cell incubation with [C^14^] radiolabeled ADP. As depicted in Figure 4D, supTh17 and iT-reg were both able to generate adenosine that supTh17 cells further effectively degraded into inosine. In contrast, Th17 cells were capable of hydrolyzing ADP into AMP but did not generate extracellular adenosine, in accordance with low levels of CD39 and CD73 expression. In keeping with concomitant CD39 and CD73 expression, supTh17 are therefore competent in generating adenosine, which is then effectively degraded into inosine.

### Effects of Adenosinergic Signaling on Cell Phenotype and Function

**Figure 5:**
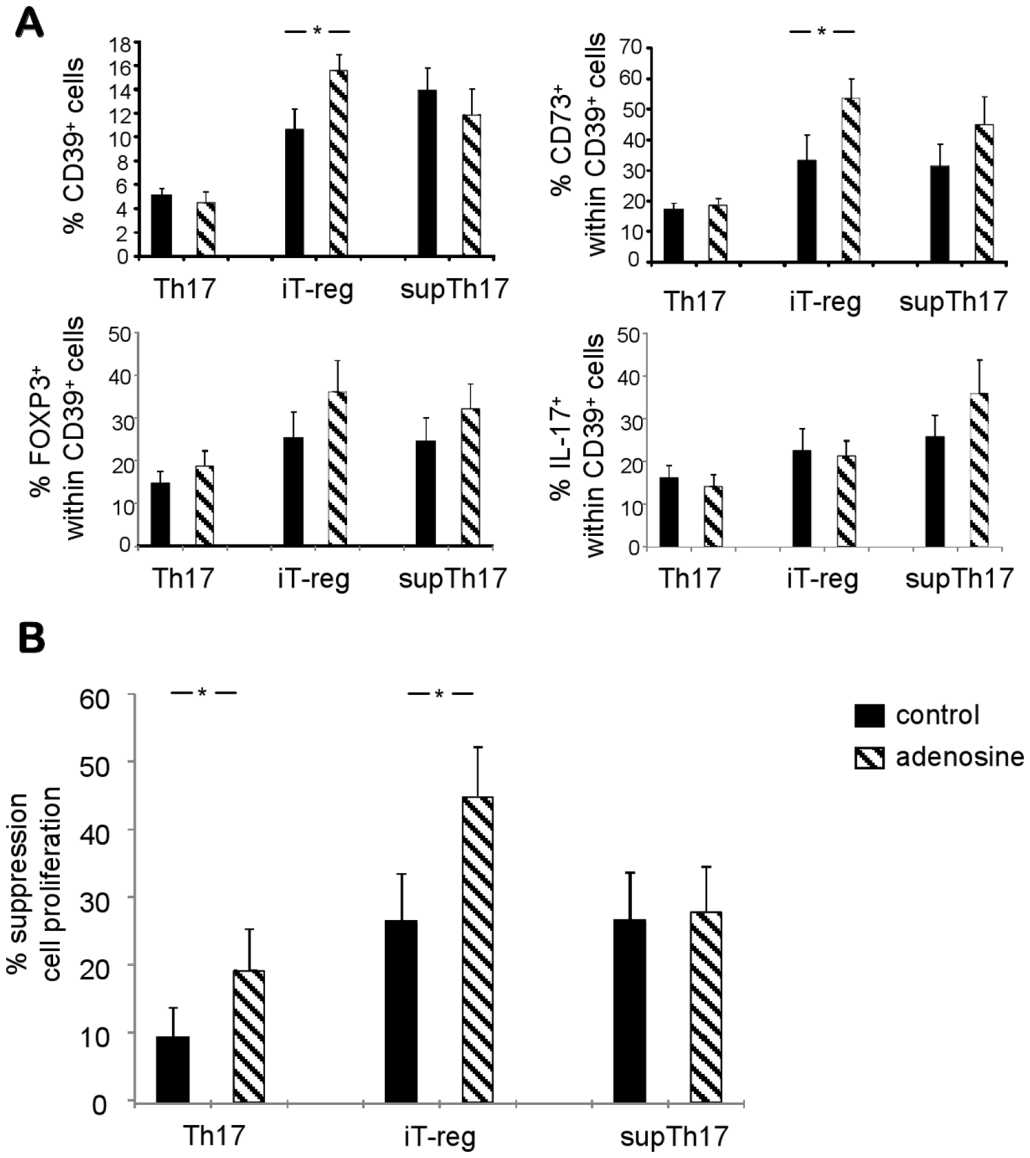
Adenosinergic effects on cell immune phenotype and function. (A) Mean (+SEM) frequency of CD39^+^ cells and of CD73^+^, FOXP3^+^ and IL-17^+^ lymphocytes within them in CD4^mem^ at baseline, Th17, iT-reg and supTh17. Results from n = 12 healthy subjects. (B) Mean (+SEM) inhibition of CD4 T-cell proliferation by Th17, iT-reg and supTh17 in the absence or presence of adenosine. Adenosine boosts expression of CD39 and CD73 and enhances the suppressor properties of iT-reg, while not having any effect on supTh17. *P≤0.05.

We then tested the effect of adenosine exposure on supTh17 and compared with that in iT-reg and Th17 cells. Adenosine increased the frequency of CD39^+^ and CD73^+^ cells among iT-reg while not having any effect on the frequency of these cells among Th17 and supTh17 cells (Figures 5A and S7 and data not shown). Exposure to exogenous adenosine did not affect the proportion of FOXP3^+^ and IL-17^+^ cells in any of the three cell subsets (Figure 5A). With regard to suppressive function, adenosine enhanced the ability of iT-reg and, though to a lesser extent, Th17 cells to control CD4 target cell proliferation while not having any effect on the suppression exerted by supTh17 (Figure 5B). The above data show that adenosine boosts the phenotypic and functional properties of iT-reg while not having any effect on supTh17.

**Figure 6:**
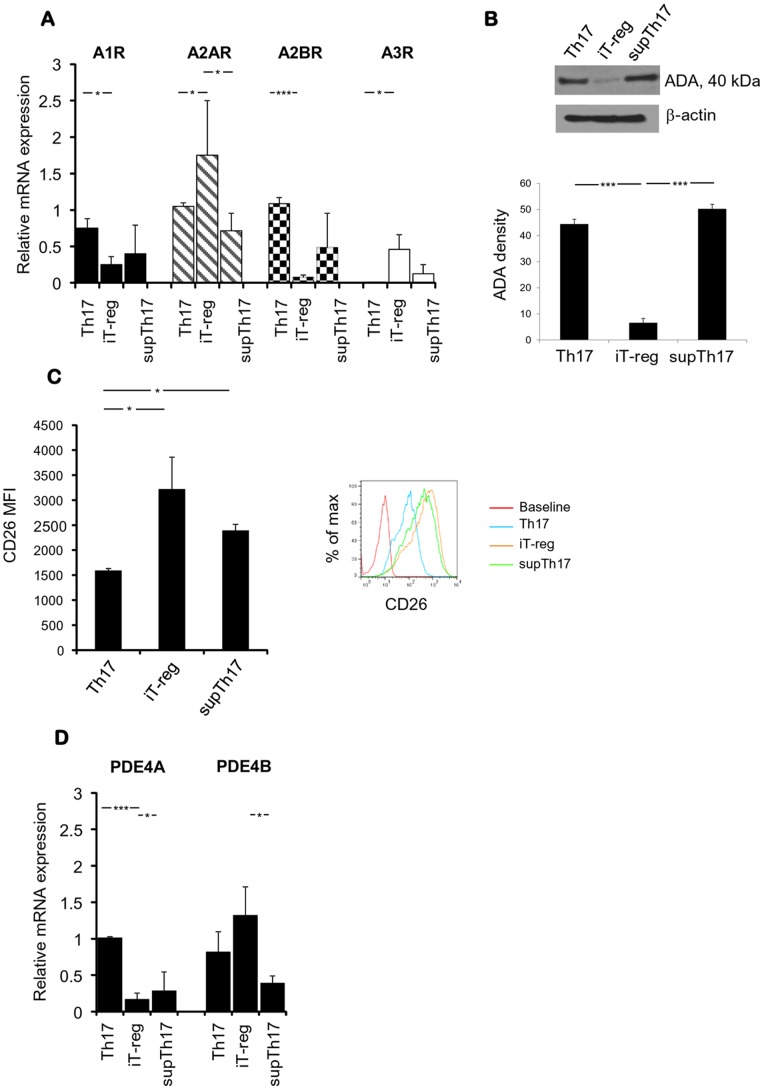
Purinergic molecular signatures of supTh17 cells. (A) Relative mRNA expression of A1, A2A, A2B, A3 receptors by Th17, iT-reg and supTh17 was determined by quantitative real-time PCR in 10 healthy subjects. Results are expressed as mean+SEM. (B) Expression of ADA was determined by immunoblot analysis. One representative of 3 independent experiments is shown. Mean (+SEM) ADA densities noted in Th17, iT-reg and supTh17 cells are also shown. (C) Mean (+SEM) CD26 MFI in Th17, iT-reg and supTh17 cells obtained from 5 healthy subjects was evaluated by flow cytometry. A representative histogram of CD26 fluorescence in CD4^mem^ at baseline, Th17, iT-reg and supTh17 is shown. (D) Mean (+SEM) relative mRNA expression of PDE4A and PDE4B was determined by quantitative real-time PCR in 10 healthy subjects. supTh17 uniquely express low levels of A2A adenosine receptor, exhibit ADA activity associated with CD26 but do not substantially up-regulate levels of PDE. *P≤0.05; ***P≤0.001.

We then examined possible mechanisms that could further account for resistance of supTh17 to exogenous adenosine. We considered that resistance to adenosine may result from low expression levels of adenosine receptors, from high levels of adenosine deaminase (ADA), which degrades adenosine into inosine, and/or high expression of phosphodiesterases (PDE) - the enzymes degrading the phosphodiester bond of cAMP. Thus, we determined the expression of A1, A2A, A2B and A3 adenosine receptors by quantitative real-time PCR. The expression of A2A receptor, known to be involved in down-regulation of inflammation and protection from tissue damage [45], was decreased at mRNA levels in supTh17, when compared to Th17 and iT-reg (Figure 6A).

In order to test whether adenosine resistance of supTh17 was the result of enhanced adenosine clearance, we first assessed the expression of ADA. We observed expression of ADA in Th17 and supTh17 (Figure 6B), indicating that both these cell types have the ability to deaminate adenosine. In contrast, ADA was only weakly expressed in iT-reg (Figure 6B). ADA is completely functional at the cell surface (known as ecto-ADA), where it directly interacts with the dipeptidylpeptidase IV (CD26) and regulates adenosine receptors. ADA activity depends on CD26, the expression of which has been recently reported to be increased on human Th17 [46]. We therefore assessed the expression of CD26 and found that the CD26 MFI was higher in iT-reg and supTh17 compared to Th17 (Figure 6C). These data indicate that the effective degradation of adenosine into inosine displayed by supTh17 relies on the co-expression of both ADA and CD26. In contrast Th17 and iT-reg, which express either ADA (Th17) or CD26 (iT-reg), do not display effective deamination activity (Figure 6C).

We next determined the expression of PDE4A and PDE4B. We found that both enzymes are expressed by Th17, iT-reg and supTh17 (Figure 6D). The supTh17, however, did not overexpress any of the PDE, ruling out the possibility that the adenosine resistance noted in these cells result from high levels of cAMP clearance. We therefore conclude that supTh17 resistance to exogenous adenosine is associated with low A2A adenosine receptor expression and enhanced scavenging of nucleosides by ecto-adenosine deaminase.

### Demonstration of supTh17 in Healthy Subjects and in Patients with Crohn’s Disease

To investigate the biological relevance of supTh17, the frequency of CD4^+^IL-17^+^ and that of supTh17 was determined in PBMCs and LPMCs obtained from healthy subjects and Crohn’s patients. These supTh17 were identified by initially gating CD4^+^CD45RO^+^ cells within PBMCs or LPMCs and then by determining the proportion of CD39^+^IL-17^+^ and FOXP3^+^ within this population.

**Figure 7:**
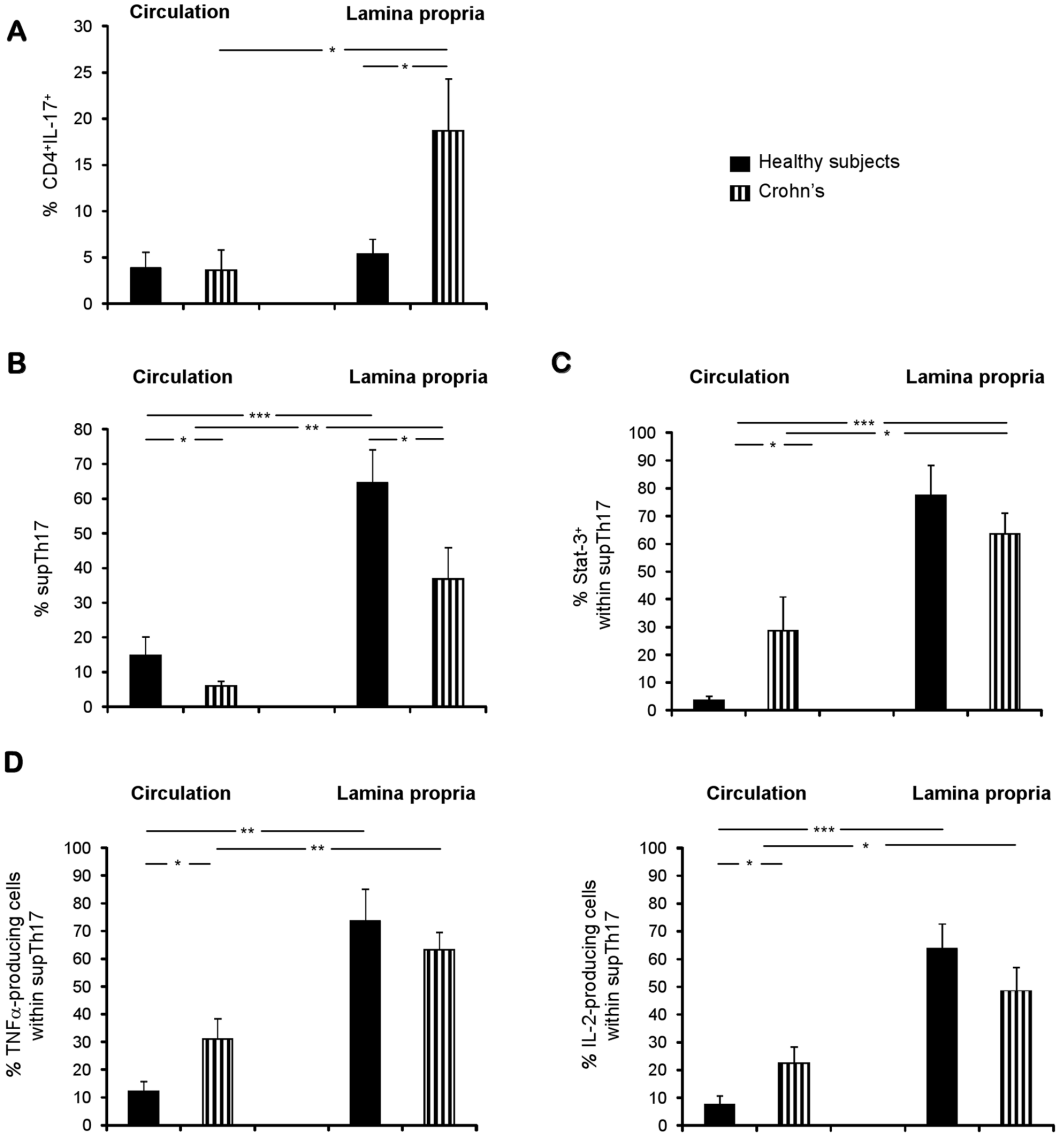
Demonstration of supTh17 cells in healthy subjects and associated decreases in Crohn’s disease. The frequency of CD4^+^IL-17^+^ and of supTh17 was determined in PBMCs and LPMCs by flow cytometry. supTh17 were identified by initially gating CD4^+^CD45RO^+^ cells within PBMCs or LPMCs and then by determining the proportion of CD39^+^IL-17^+^ and FOXP3^+^ within them. Mean (+SEM) frequency of (A) CD4^+^IL-17^+^ and of (B) supTh17 cells in the circulation and in the lamina propria. Mean (+SEM) frequency of supTh17 positive for (C) Stat-3 and for (D) TNF-α and IL-2 in the circulation and in the lamina propria. Healthy subjects: n = 17; Crohn’s: n = 25; *P≤0.05; **P≤0.01; ***P≤0.001.

While the proportion of CD4^+^IL-17^+^ in PBMCs cells was similar in the two groups, that of CD4^+^IL-17^+^ lymphocytes obtained from the lamina propria was higher in Crohn’s patients than in healthy subjects (Figure 7A). In patients, the frequency of CD4^+^IL-17^+^ cells was markedly higher in the lamina propria compared to the circulation (Figure 7A). We then determined the frequency of supTh17 in both compartments. These supTh17 were decreased in Crohn’s patients, when compared to healthy subjects, both within PBMC and LPMC populations (Figures 7B and S8). In both groups, supTh17 were increased in the lamina propria compared to the circulation (Figures 7B and S8). When analyzed for expression of Stat-3 known to modulate Th17 immunosuppressive activity through up-regulation of CD39 [27] - circulating supTh17 from Crohn’s patients displayed higher proportion of cells positive for this marker than did comparably prepared cells from healthy subjects (Figure 7C) [27]. In both groups, supTh17 from the lamina propria contained higher proportions of lymphocytes that were positive for Stat-3 (Figure 7C). Analysis of cytokine profiles show that supTh17 from Crohn’s disease patients had higher frequencies of TNF-α^+^ and IL-2^+^ cells - pro-inflammatory cytokines previously reported to be decreased in populations of Th17 cells with suppressive properties isolated from the small intestine [47], than the respective supTh17 from healthy subjects in the circulation (albeit not in the lamina propria; Figure 7D). In both groups, supTh17 in the lamina propria contained higher proportions of TNF-α^+^ and IL-2^+^ cells than did the counterparts in the circulation (Figure 7D). No differences in the frequencies of supTh17 and of TNF-α^+^ and IL-2^+^ cells within them were noted between patients with either active or inactive disease. The above data indicate that supTh17 are highly represented in the lamina propria and that the frequencies of these cells are lower in the periphery in Crohn’s disease, where in contrast these cells also express increased levels of pro-inflammatory cytokines.

## Discussion

We have shown that a population of human supTh17 cells can be derived following the exposure of iT-reg to Th17 polarizing conditions *in vitro*. These putative supTh17 display phenotypic features of both effector Th17 (i.e. production of IL-17 and expression of CCR6, IL-22 and IL-23R) and iT-reg (expression of FOXP3) and importantly control effector cell function by inhibiting CD4 cell proliferation as well as production of IFNγ and IL-17. It is not clear whether these cells might be representative of a late stage in Th17 differentiation, in which the effector potential of prototypic Th17 cells is attenuated or rather constitute unique cell subsets in which overlapping regulatory and effector features coexist.

The robust *in vitro* system used in the present study enabled us to observe changes in T-cell phenotype and function upon stimulation in the presence of Th17 and iT-reg polarizing conditions. Previous studies have documented differentiation of CD4 cells into Th17 or iT-reg following short- and medium-term cell culture *in vitro* [17], [18], [48], [49]. These and our studies may be of particular relevance to disease settings, in which antigen-primed CD4 memory cells may be sequentially exposed to different cytokine milieus and undergo modulation accordingly, during either inflammatory or remission phases.

Given the putative importance of CD39 in immunoregulation, particularly concerning purinergic mechanisms governing the suppressive function of iT-reg, we studied whether supTh17 expressed this ectonucleotidase. Our data show that, in contrast to prototypic Th17 cells, supTh17 display high levels of CD39. Furthermore, supTh17 cells also co-express ecto-5′-ectonucleotidase CD73, which is pivotal in the generation of adenosine from AMP. These supTh17, in contrast to prototypic Th17, have the potential to generate adenosine in a manner comparable to iT-reg, which can be noted by standard biochemical tests (Figure 4D). However, in a manner distinct from iT-reg, the extracellular adenosine that is generated by supTh17 undergoes further degradation, given the concomitant expression of adenosine deaminase and CD26 by these cells. In accordance with the low CD73 levels expressed, prototypic Th17 cells were unable to generate adenosine.

When we examined the effect of exogenous adenosine on supTh17 phenotypic and functional properties, we could observe that these cells were resistant to the effect of this mediator. Curiously, these cells did not undergo upregulation of CD39 expression nor did these cells exhibit amelioration of suppressive function, in the manner observed in anergic type iT-reg.

Adenosine resistance in supTh17 cells is likely to be conferred by low levels of A2A adenosine receptor and by higher levels of adenosine catalysis. The A2A adenosine receptor is primarily known to mediate anti-inflammatory effects: lymphocytes from A2A receptor (−/−) mice show higher rates of cell proliferation and produce high IFNγ levels upon stimulation [50]. A2A receptor stimulation has established inhibitory effects on Th1 and Th17 effector cell generation and, in contrast, favors generation of FOXP3^+^ and LAG-3^+^ regulatory T-cells [51]. Our data suggest that the most likely mechanisms for supTh17 resistance to adenosine are linked to low A2A receptor levels and enhanced levels of adenosine catalysis, enabled by ADA and CD26 co-expression. We have observed that iT-reg display marked decreases in mRNA levels of the A2B adenosine receptor. This observation might have relevance for the differential effects of A2B versus A2A signaling by these cells. Furthermore, Moriyama and Sitkovsky have demonstrated in studies of A2AR versus A2BR expression in transfected cells that substantive proportions of A2BR are preferentially degraded by the proteasome, a mechanism that might be also operative here in differentiating Th17 cells from iT-reg [52].

**Figure 8:**
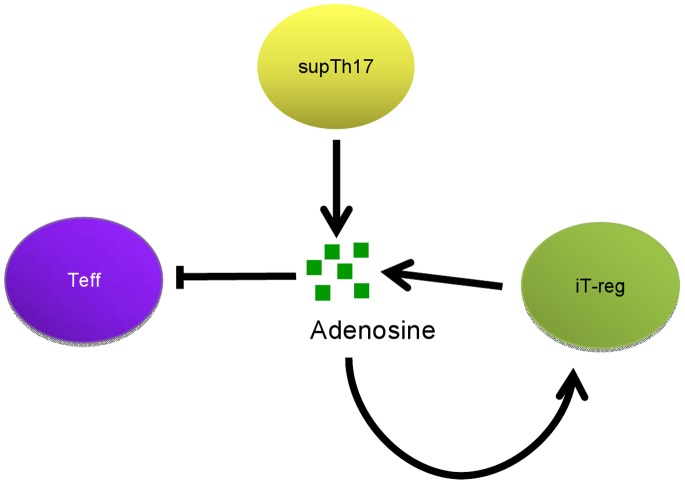
SupTh17, iT-reg and purinergic control of T-cell immune responses. Both supTh17 and iT-reg cells have the capacity to suppress effector T-cells (Teff) by generating adenosine. In a manner distinct from iT-reg which are anergic, however, supTh17 express low levels of A2A receptor and exhibit nucleoside scavenging ecto-enzymatic activity. These properties confer on supTh17 an important intrinsic resistance to suppressive effects of adenosine, which may develop in parallel with prolonged cellular activation in accordance with memory T-cell status. These differences suggest that supTh17 might undergo conversion and be recruited as suppressor-type cells in the later evolution of immune responses where these cells may persist at sites of resolving injury.

The observation that supTh17 are resistant to adenosinergic modulation implies that these cells are not conventional suppressors, nor are these cells anergic. The supTh17 cells might adapt their own intrinsic ability to regulate or inflict damage according to the immunological context in which they operate. Given that in these cells, both regulatory (i.e. adenosine generation, suppressive function) and pro-inflammatory (i.e. low levels of A2A and inosine generation) features co-exist, it is plausible to suggest that supTh17 might reside in a form of ‘purinergic limbo’ and unresponsiveness until a crucial time where this balance may be perturbed. This temporal ‘status’ would enable supTh17 cells to influence extrinsic homeostatic properties of target cells via suppression mediated through generation of adenosine while maintaining intrinsic resistance to this immune suppressive molecule. In contrast, iT-reg exert suppression via production of adenosine, while being also wholly susceptible to the nucleoside modulatory effects that might stabilize their immune suppressive phenotype (Figure 8; [4], [31]). At variance with iT-reg, supTh17 are not subjected to this autocrine loop, suggesting that these cells, in contrast, may play roles as both regulators of late stage immune responses and in the maintenance of T-cell memory functionality.

Another important finding of this investigation is that supTh17 could be enumerated in both circulation and lamina propria of healthy subjects and patients with Crohn’s disease. These cells appear to preferentially home to the intestine, as demonstrated by their high percentages in the lamina propria, suggesting that the intestine may be the compartment where Th17 cells undergo regulation. Our data indicate there are higher percentages of supTh17 cells expressing Stat-3 in the lamina propria indicating that this transcription factor may have a role in the expression of CD39 and induction of supTh17 in the colon [27]. In agreement with these data, in mouse models of colitis, pathogenic Th17 cells are also considered to undergo regulation in the intestine where these cells acquire phenotypic and functional T-reg-like properties [47].

Importantly, supTh17 numbers are markedly decreased in Crohn’s patients. This decrease might theoretically result in disease exacerbation and perpetuation because of the decreased ability of effector Th17 to undergo regulation. Previous clinical studies demonstrated impaired immunoregulation and particularly numerically defective and dysfunctional T-reg in these same disease settings [53], [54].

Hence, the increased numbers of effector Th17 cells, also shown here may originate from defective control usually operated by active immune suppression - i.e. primarily defective T-reg, or alternatively be the result of decreased Th17 autoregulation. Interestingly, supTh17 from Crohn’s patients appear skewed towards a pro-inflammatory phenotype as these cells contain higher frequencies of TNF-α and IL-2 pro-inflammatory cytokines than those noted in healthy controls.

In conclusion, we have shown that human supTh17 can be obtained upon exposure of iT-reg to Th17 driving conditions *in vitro*.

High levels of CD39 expression distinguish these immune suppressive cells from effector pathogenic Th17 cells. We propose that these fundamental alterations in purinergic signaling might control tissue damage while limiting cellular pathogenicity in local and systemic inflammatory illnesses, such as in Crohn’s disease. Promoting the local expansion of supTh17 cells and the maintenance of these should boost local immune suppressive activities and augment diminished T-reg functionality, as previously noted in IBD [34,55]. Indeed, these studies and development of modalities to boost CD39 expression have implications for the development of novel therapeutic strategies in Crohn’s disease.

## Supporting Information

Figure S1 **Experimental protocol for T-cell activation.** CD4^mem^ and CD4^naive^ T-cells, purified as CD4^+^CD45RO^+^ and CD4^+^CD45RA^+^ cells, were initially activated under Th17 polarizing conditions. This comprised of IL-6+IL-1β+rTGF-β+anti-CD3/anti-CD28 T-cell expander (bead/cell ratio: 1∶50) for 3 days. Cells were then exposed to iT-reg skewing conditions with high concentration IL-2 and anti-CD3/anti-CD28 T-cell expander (bead/cell ratio: 1∶2) for 4 days, and then were re-activated under Th17 polarizing conditions for 3 days.
(TIF)

Figure S2 **Frequency of IL-17^+^, CD25^+^ and FOXP3^+^ cells in Th17, iT-reg and supTh17.** Frequency of (A) IL-17^+^, (B) CD25^+^ and (C) FOXP3^+^ cells in CD4^mem^ at baseline, Th17, iT-reg and supTh17 cells was determined in 12 healthy subjects; ***P<0.001.
(TIFF)

Figure S3 **iT-reg phenotype.** Flow cytometry plots of FOXP3 (*X* axis) and IFNγ or IL-2 (*Y* axis) fluorescence. Frequency of cells is shown in each quadrant. A representative of two independent experiments is shown.
(TIFF)

Figure S4 **T-cell suppressive ability.** (A) Mean (+SEM) CD4 effector cell proliferation, expressed as mean count per minute (cpm) in the absence or presence of Th17, iT-reg and supTh17 cells. Proliferation of Th17, iT-reg and supTh17 on their own is also shown. (B) Mean (+SEM) CD4 effector cell IL-17 and IFNγ production in the absence or presence of Th17, iT-reg and supTh17 cells. Production of IL-17 and IFNγ by Th17, iT-reg and supTh17, in isolation, are also shown. Results are obtained from 10 healthy subjects. *P≤0.05; **P≤0.01; ***P<0.001. (C) Representative flow cytometry plots of CD4 (*X* axis) and IL-17 or IFNγ (*Y* axis) fluorescence in CD4 effectors alone and in the presence of Th17, iT-reg or supTh17 cells.
(TIF)

Figure S5 **Frequency of CD39^+^ and CD73^+^ cells within Th17, iT-reg and supTh17.** (A) Frequency of CD39^+^ cells was determined after exposing CD4^mem^ cells to different Th17 polarizing conditions, i.e. 1) IL-6+IL-1β+rTGF-β; 2) IL-6+IL-1β+IL-23; and 3) IL-6+IL-1β+rTGF-β+IL-23. Flow cytometry plots of CD4 (*X* axis) and CD39 (*Y* axis) fluorescence. A representative of 5 independent experiments is shown. (B) Flow cytometry plots of CD4 (*X* axis) and CD73 (*Y* axis) fluorescence. Cells were gated on CD39^+^ lymphocytes.
(TIFF)

Figure S6 **Phenotype of Th1, iT-reg and supTh17 cells.** Mean (+SEM) frequency of lymphocytes positive for (A) FOXP3, (B) IL-10 and (C) RORC within CD39^+^ cells in CD4^mem^ at baseline, Th17, iT-reg and supTh17. Results are obtained from 12 healthy subjects. *P≤0.05; **P≤0.01. Representative flow cytometry plots of CD4 (*X* axis) and (A) FOXP3, (B) IL-10 and (C) RORC (*Y* axis) fluorescence in CD4^mem^ at baseline, Th17, iT-reg and supTh17 are shown. Cells are gated on CD39^+^ lymphocytes.
(TIF)

Figure S7 **Effect of adenosine on CD39 expression.** Flow cytometry plots of CD4 (*X* axis) and CD39 (*Y* axis) fluorescence in Th17, iT-reg and supTh17 cells in the absence and presence of adenosine in a representative individual of 12 healthy subjects tested.
(TIFF)

Figure S8 **Frequency of supTh17 in PBMCs and LPMCs.** supTh17 were identified by initially gating CD4^+^CD45RO^+^ cells within PBMCs or LPMCs and then by determining the proportion of cells positive for CD39 and IL-17 and expressing FOXP3 within this population. Flow cytometry plots of CD4 (*X* axis) and IL-17 (*Y* axis) fluorescence in PBMCs and LPMCs from one healthy subject and one patient with Crohn’s disease. Cells were gated on CD39^+^ lymphocytes. Histograms of FOXP3 fluorescence in CD4^+^IL-17^+^ cells within CD39^+^ lymphocytes are also shown.
(TIFF)
