# Supplementary figures and images for: Characterization of Human CD39+ Th17 Cells with Suppressor Activity and Modulation in Inflammatory Bowel Disease
Source: PLoS One. 2014 Feb 5;9(2):e87956. doi: 10.1371/journal.pone.0087956 (PMC3914873; doi:10.1371/journal.pone.0087956)

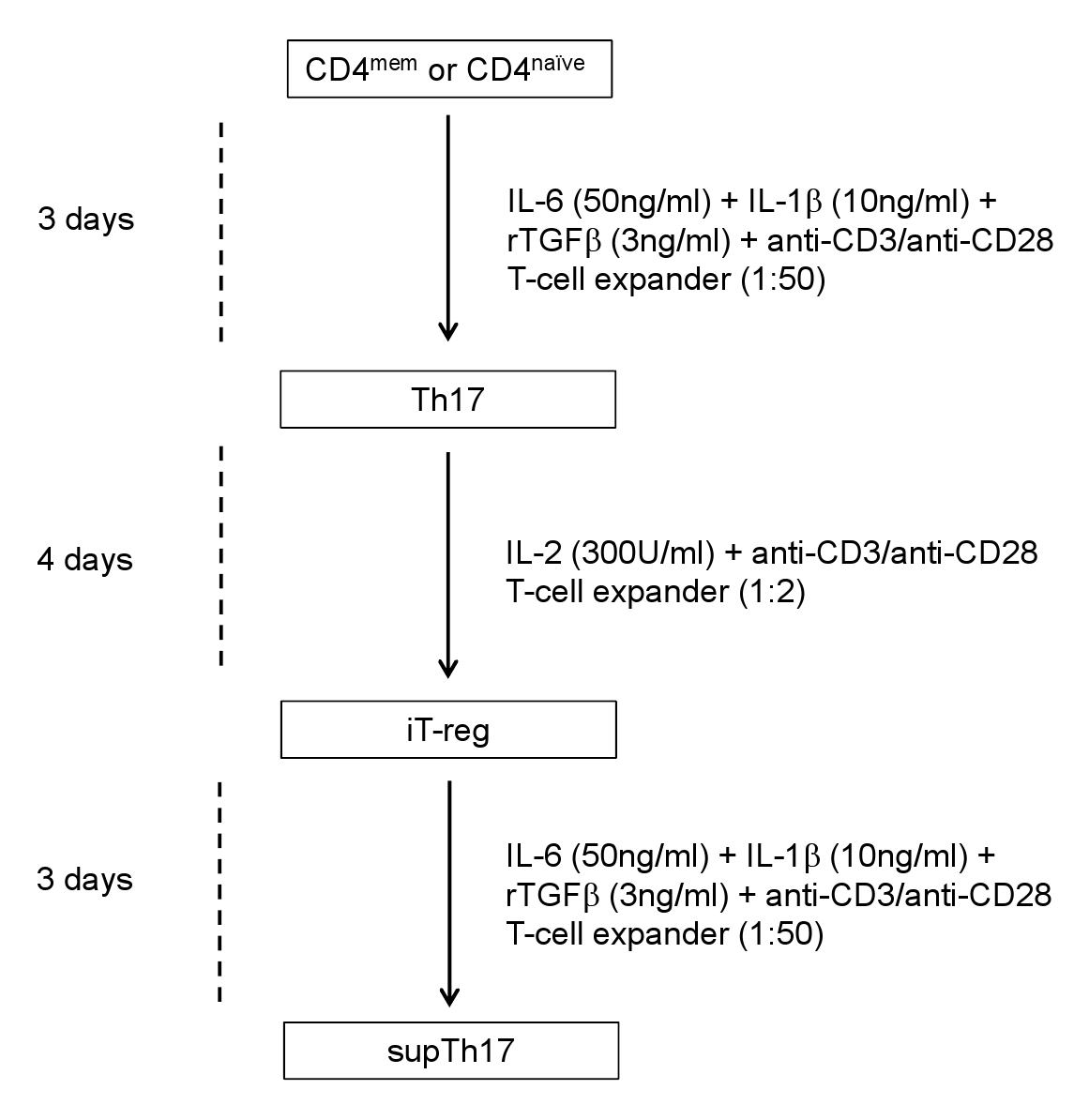

Supplement: Figure S1 — Experimental protocol for T-cell activation. CD4mem and CD4naive T-cells, purified as CD4+CD45RO+ and CD4+CD45RA+ cells, were initially activated under Th17 polarizing conditions. This comprised of IL-6+IL-1β+rTGF-β+anti-CD3/anti-CD28 T-cell expander (bead/cell ratio: 1∶50) for 3 days. Cells were then exposed to iT-reg skewing conditions with high concentration IL-2 and anti-CD3/anti-CD28 T-cell expander (bead/cell ratio: 1∶2) for 4 days, and then were re-activated under Th17 polarizing conditions for 3 days. (TIF) [file pone.0087956.s001.tif]

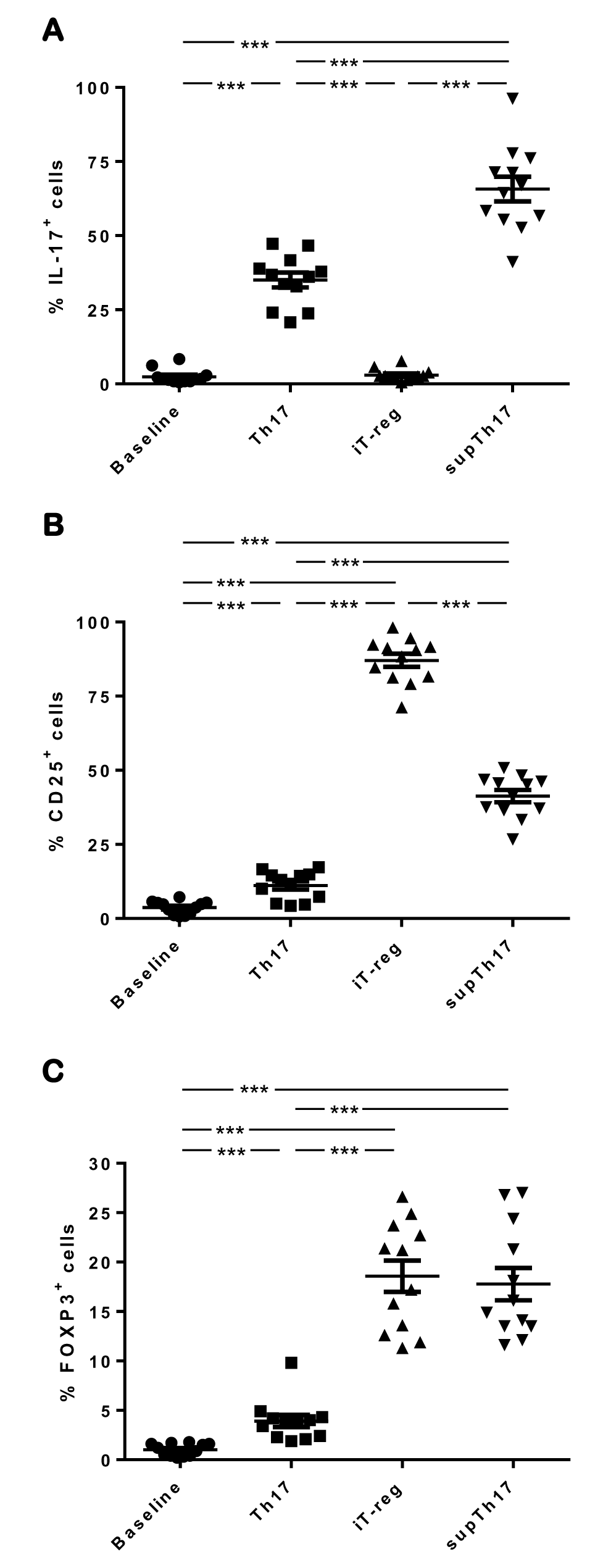

Supplement: Figure S2 — Frequency of IL-17+, CD25+ and FOXP3+ cells in Th17, iT-reg and supTh17. Frequency of (A) IL-17+, (B) CD25+ and (C) FOXP3+ cells in CD4mem at baseline, Th17, iT-reg and supTh17 cells was determined in 12 healthy subjects; ***P<0.001. (TIFF) [file pone.0087956.s002.tiff]

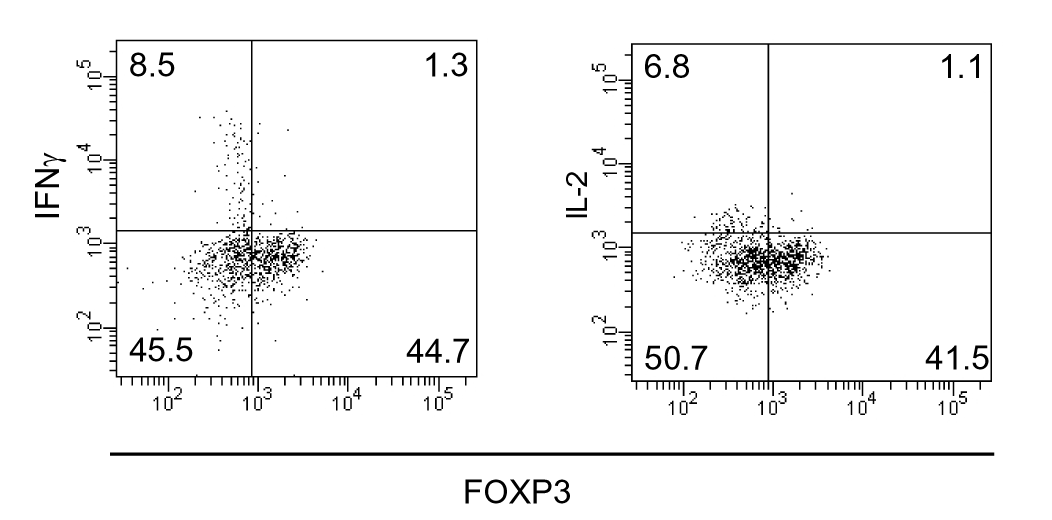

Supplement: Figure S3 — iT-reg phenotype. Flow cytometry plots of FOXP3 (X axis) and IFNγ or IL-2 (Y axis) fluorescence. Frequency of cells is shown in each quadrant. A representative of two independent experiments is shown. (TIFF) [file pone.0087956.s003.tiff]

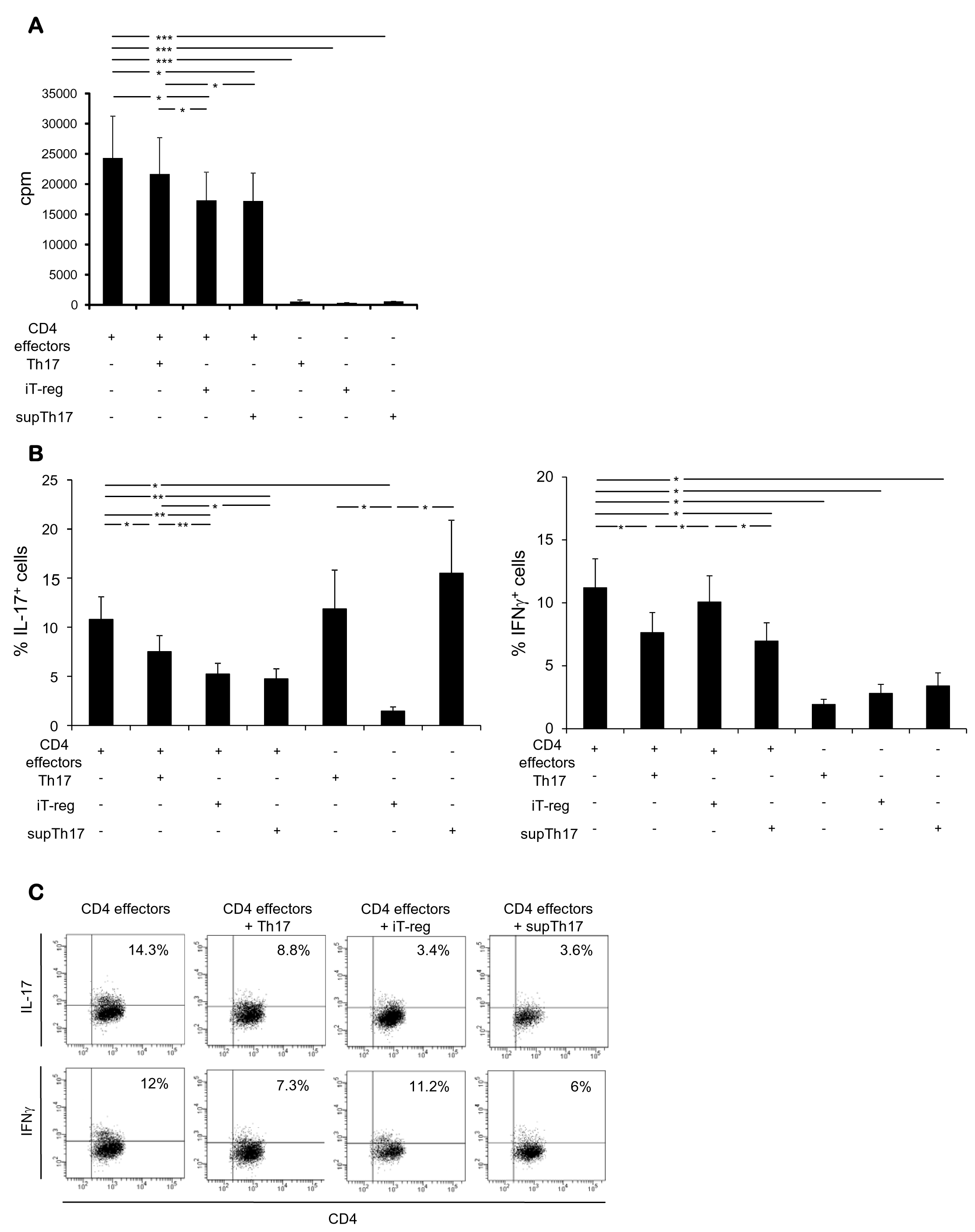

Supplement: Figure S4 — T-cell suppressive ability. (A) Mean (+SEM) CD4 effector cell proliferation, expressed as mean count per minute (cpm) in the absence or presence of Th17, iT-reg and supTh17 cells. Proliferation of Th17, iT-reg and supTh17 on their own is also shown. (B) Mean (+SEM) CD4 effector cell IL-17 and IFNγ production in the absence or presence of Th17, iT-reg and supTh17 cells. Production of IL-17 and IFNγ by Th17, iT-reg and supTh17, in isolation, are also shown. Results are obtained from 10 healthy subjects. *P≤0.05; **P≤0.01; ***P<0.001. (C) Representative flow cytometry plots of CD4 (X axis) and IL-17 or IFNγ (Y axis) fluorescence in CD4 effectors alone and in the presence of Th17, iT-reg or supTh17 cells. (TIF) [file pone.0087956.s004.tif]

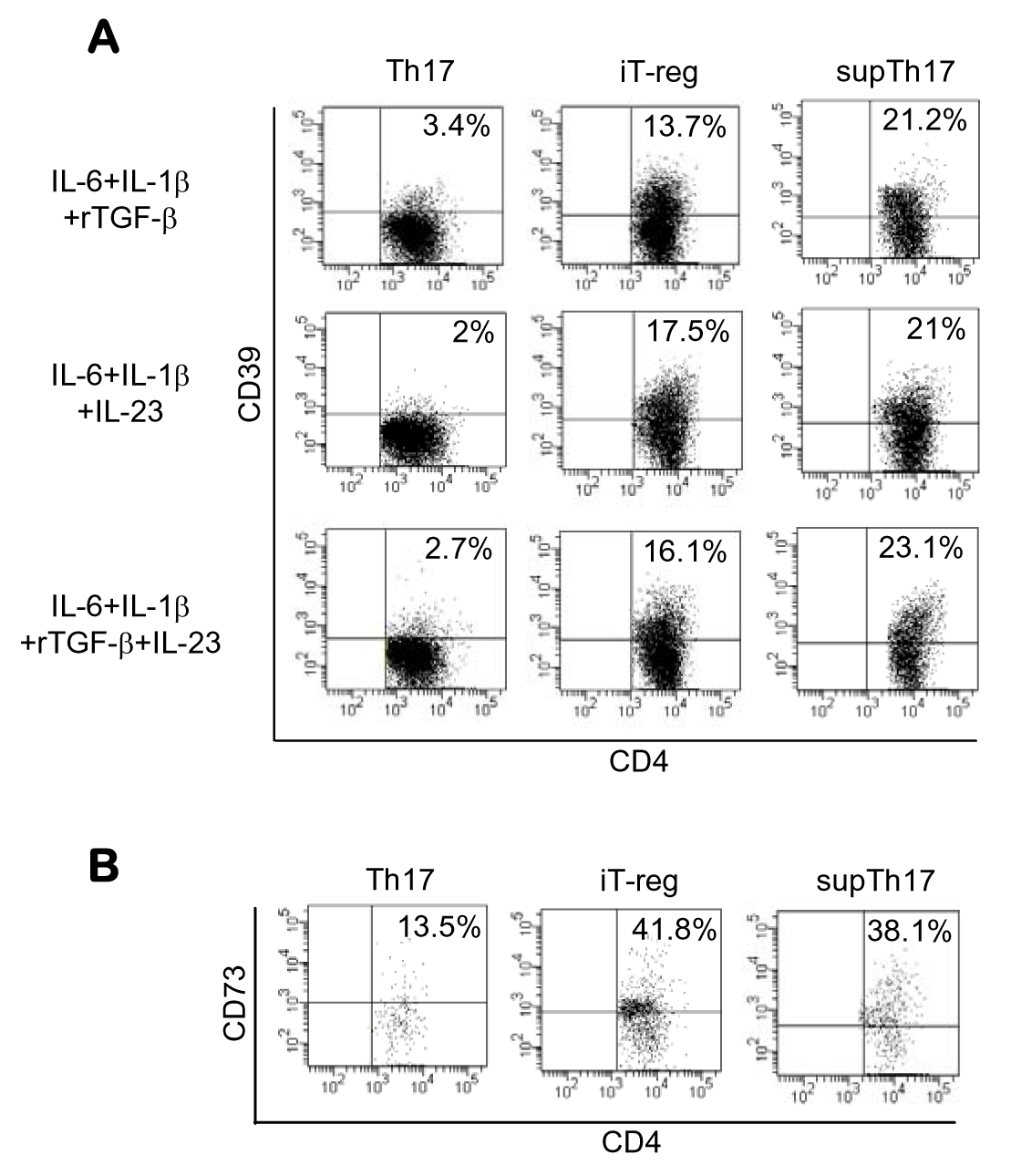

Supplement: Figure S5 — Frequency of CD39+ and CD73+ cells within Th17, iT-reg and supTh17. (A) Frequency of CD39+ cells was determined after exposing CD4mem cells to different Th17 polarizing conditions, i.e. 1) IL-6+IL-1β+rTGF-β; 2) IL-6+IL-1β+IL-23; and 3) IL-6+IL-1β+rTGF-β+IL-23. Flow cytometry plots of CD4 (X axis) and CD39 (Y axis) fluorescence. A representative of 5 independent experiments is shown. (B) Flow cytometry plots of CD4 (X axis) and CD73 (Y axis) fluorescence. Cells were gated on CD39+ lymphocytes. (TIFF) [file pone.0087956.s005.tiff]

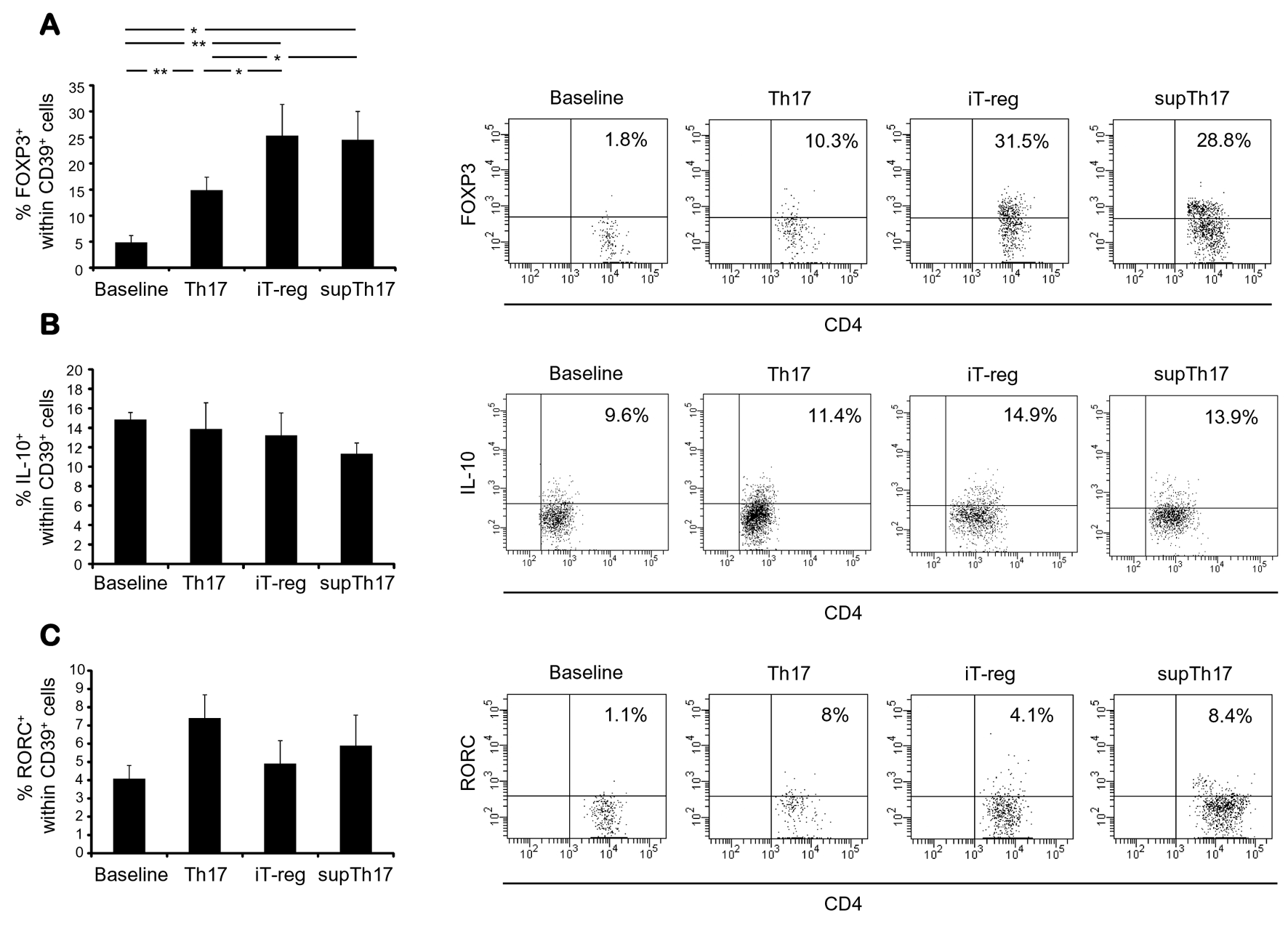

Supplement: Figure S6 — Phenotype of Th1, iT-reg and supTh17 cells. Mean (+SEM) frequency of lymphocytes positive for (A) FOXP3, (B) IL-10 and (C) RORC within CD39+ cells in CD4mem at baseline, Th17, iT-reg and supTh17. Results are obtained from 12 healthy subjects. *P≤0.05; **P≤0.01. Representative flow cytometry plots of CD4 (X axis) and (A) FOXP3, (B) IL-10 and (C) RORC (Y axis) fluorescence in CD4mem at baseline, Th17, iT-reg and supTh17 are shown. Cells are gated on CD39+ lymphocytes. (TIF) [file pone.0087956.s006.tif]

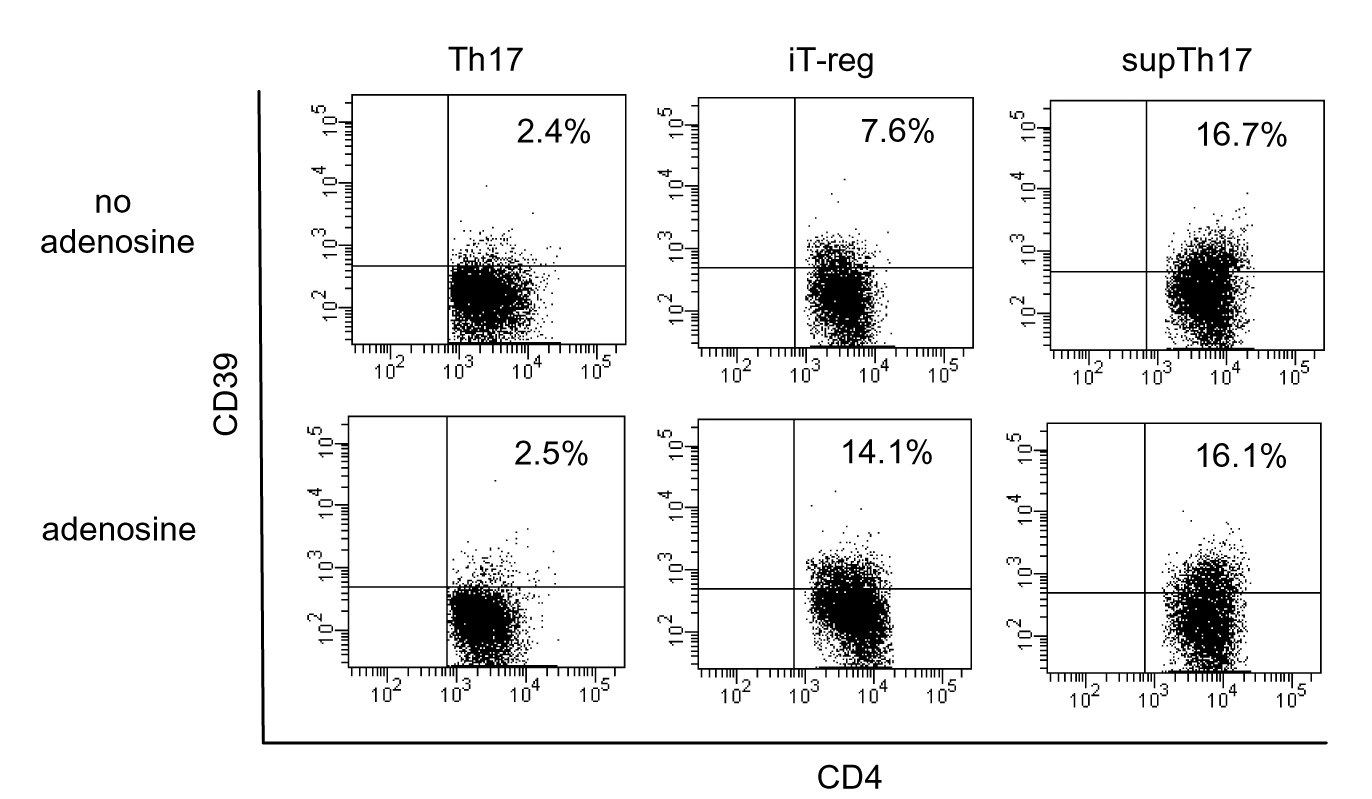

Supplement: Figure S7 — Effect of adenosine on CD39 expression. Flow cytometry plots of CD4 (X axis) and CD39 (Y axis) fluorescence in Th17, iT-reg and supTh17 cells in the absence and presence of adenosine in a representative individual of 12 healthy subjects tested. (TIFF) [file pone.0087956.s007.tiff]

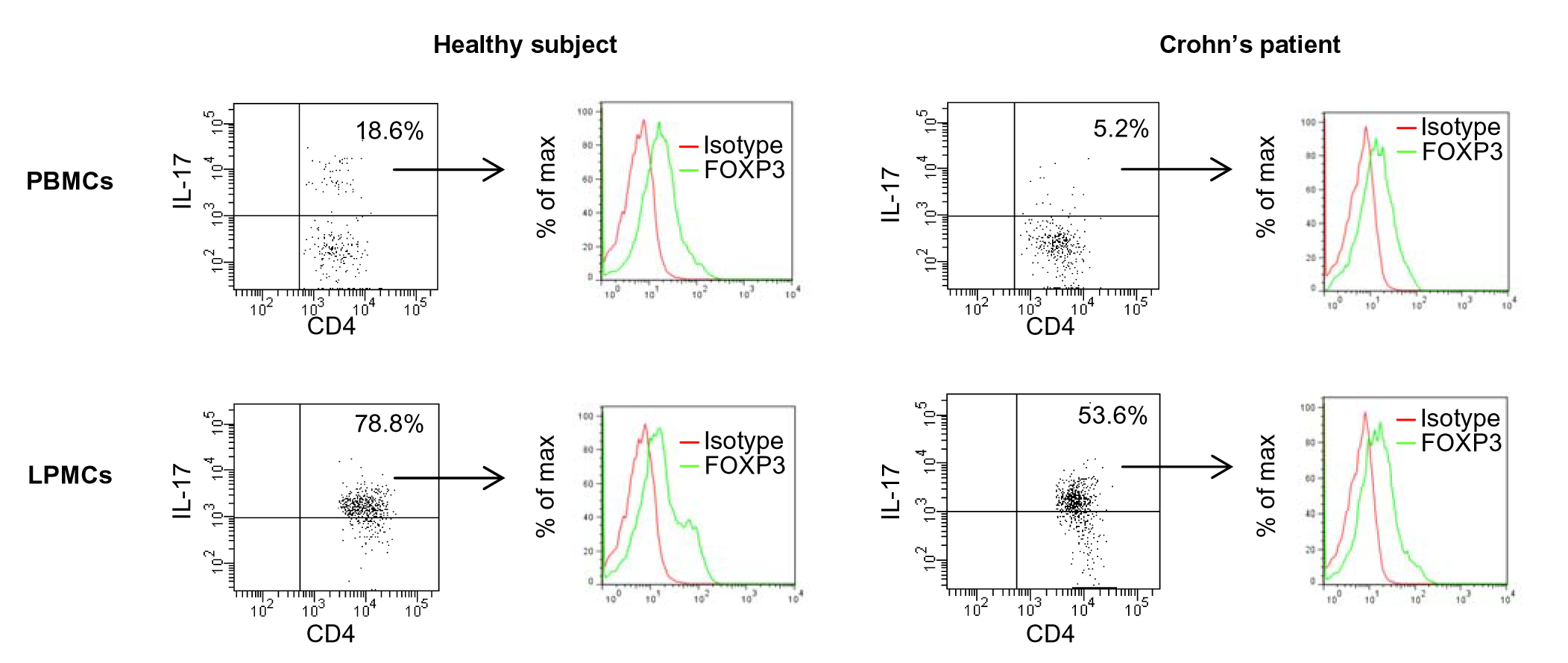

Supplement: Figure S8 — Frequency of supTh17 in PBMCs and LPMCs. supTh17 were identified by initially gating CD4+CD45RO+ cells within PBMCs or LPMCs and then by determining the proportion of cells positive for CD39 and IL-17 and expressing FOXP3 within this population. Flow cytometry plots of CD4 (X axis) and IL-17 (Y axis) fluorescence in PBMCs and LPMCs from one healthy subject and one patient with Crohn’s disease. Cells were gated on CD39+ lymphocytes. Histograms of FOXP3 fluorescence in CD4+IL-17+ cells within CD39+ lymphocytes are also shown. (TIFF) [file pone.0087956.s008.tiff]
